# Incorporation of Locust Bean Gum and Solid Lipid Microparticles as Strategies to Improve the Properties and Stability of Calcium-Rich Soy Protein Isolate Gels

**DOI:** 10.3390/gels10070467

**Published:** 2024-07-17

**Authors:** Thais C. Brito-Oliveira, Ana Clara M. Cavini, Leticia S. Ferreira, Izabel C. F. Moraes, Samantha C. Pinho

**Affiliations:** Laboratory of Encapsulation and Functional Foods (LEnAlis), Department of Food Engineering, School of Animal Science and Food Engineering (FZEA), University of São Paulo (USP), Pirassununga 13635-900, Brazil; thacbrito@gmail.com (T.C.B.-O.); leticia2.ferreira@usp.br (L.S.F.);

**Keywords:** soy protein isolate, salt-induced gels, calcium, plant proteins, locust bean gum, emulsion-filled gels

## Abstract

The present study aimed to investigate the properties of calcium-rich soy protein isolate (SPI) gels (14% SPI; 100 mM CaCl_2_), the effects of incorporating different concentrations locust bean gum (LBG) (0.1–0.3%, *w*/*v*) to the systems and the stability of the obtained gels. Also, the incorporation of solid lipid microparticles (SLMs) was tested as an alternative strategy to improve the system’s stability and, therefore, potential to be applied as a product prototype. The gels were evaluated regarding their visual aspect, rheological properties, water-holding capacities (WHCs) and microstructural organizations. The CaCl_2_-induced gels were self-supported but presented low WHC (40.0% ± 2.2) which was improved by LBG incorporation. The obtained mixed system, however, presented low stability, with high syneresis after 10 days of storage, due to microstructural compaction. The gels’ stability was improved by SLM incorporation, which decreased the gelled matrices’ compaction and syneresis for more than 20 days. Even though the rheological properties of the emulsion-filled gels (EFGs) were very altered due to the ageing process (which may affect the sensory perception of a future food originated from this EFG), the incorporation of SLMs increased the systems potential to be applied as a calcium-rich product prototype.

## 1. Introduction

The food industry is constantly facing challenges to adapt their formulations to a large variety of global changes and demands. Recently, the biggest issues of the sector are related to the global population increase (projected to achieve 9 billion by 2050), increase in life expectancy, concerns regarding the use of animal-based products and awareness of the importance of using sustainable ingredients [1,2]. In response to these tendencies, investigations regarding the functional properties of plant proteins have been growing significantly [3,4]. Most of the studies point out the challenges of applying plant proteins due to their distinct techno-functional properties in comparison to animal ingredients [5,6].

Soy protein isolate (SPI), for example, is the most important plant protein ingredient, known for its high availability in the market, high nutritional value and affordability, but also for presenting poor solubility, which affects many other functionalities, including its gelling capacity [7,8,9,10,11]. This fact was observed in a previous investigation by our research group, in which SPI was applied to produce NaCl-induced gels, and self-supported systems could be obtained only using relatively high protein concentrations (14%) [7]. Also, the strength and stability of the gels were not satisfactory, requiring the incorporation of galactomannans to improve the systems’ properties [7]. According to the literature, the incorporation of polysaccharides is one of the most interesting strategies to improve the gelling capacities of protein ingredients, as they stabilize the microstructures formed [12,13,14,15]. In fact, the incorporation of locust bean gum in soy protein gels [7] was important to produce more stable structures which was later successfully applied to produce emulsion-filled gels encapsulating beta-carotene [16].

Even though NaCl-induced gelation methods have received attention, for allowing gelation at lower protein concentrations and lower temperatures than other methods [7,17,18], the consumption of sodium has been associated with a globally high prevalence of hypertension and cardiovascular disease, especially for elderly people [19]. One alternative to overcome such problems could be the replacement of NaCl by CaCl_2_ in gel protein-based matrices.

In addition to allowing the reduction of sodium, such an alternative would increase the concentration of calcium in the potential food formulation, which is also a recent and important issue in the food industry [20,21]. According to the literature, calcium is an important raw material for bone formation, also playing important roles in other physiological activities, including coagulation, nerve and muscle activities and heart electrophysiology, among others. In this context, the maintenance of a stable extracellular calcium concentration may be seen as a homeostatic priority, especially for elderly people [20,21]. According to the literature, the World Health Organization (WHO) recommendations for calcium intake varies depending on the age, but for healthy adults is 1000 mg/day, and fortified foods can provide 30% of such recommended intake in 100 g [21].

The development of gelled food formulation with calcium and plant-based proteins is a quite complex process, requiring not only further investigations regarding the gelling capacity of SPIs and their behavior at high CaCl_2_ concentrations, but a comprehension of the stability of such gels. The stability of gels is a very important research subject, due to the highly transient and non-linear nature of such systems, which tend to present structural alterations over time, due to spontaneous processes (i.e., aging processes) and/or external forces [22,23]. Such structural rearrangements can be either subtle or more drastic, even leading to the collapse of the gels, which, for food formulations, may affect the shelf life and the acceptability of the systems [22,24,25,26]. Considering this, the quantitative prediction of stability of gels is a critical for the formulation and manufacture of commercial food products [25].

Besides polysaccharides, the properties and stability of gelled systems may be affected by the addition of lipid fillers (e.g., emulsion or solid lipid microparticles) to the matrices, originating “emulsion filled gels” (EFGs). The properties of such complex systems depend on the characteristics of the gelled matrix, the lipid fillers and interactions between such components [27,28,29]. If it presents a positive effect over the gel’s properties, the incorporation of such fillers may be interesting for future encapsulations of hydrophobic bioactive compounds, originating fortified food formulations.

Considering the promising advances of the last studies involving the cold-set gelation of soy protein isolate, but also the concerns regarding sodium consumption, the present study aimed to investigate: (i) the properties of CaCl_2_-induced gels produced using the same SPI concentration (14%) and same ionic strength (µ: 100—representing 100 mM CaCl_2_) as the ones previously applied for producing NaCl-induced gels [7], (ii) the effects of incorporating different concentrations of LBG to the systems and (iii) the stability of the obtained plant-based gel rich in calcium, and the effects of solid lipid microparticle incorporation to verify its potential application as a product prototype, for future incorporation of hydrophobic bioactives.

## 2. Results and Discussion

### 2.1. Production and Characterization of CaCl_2_-Rich Cold-Set Gels: A Comparative Discussion

**Figure 1:**
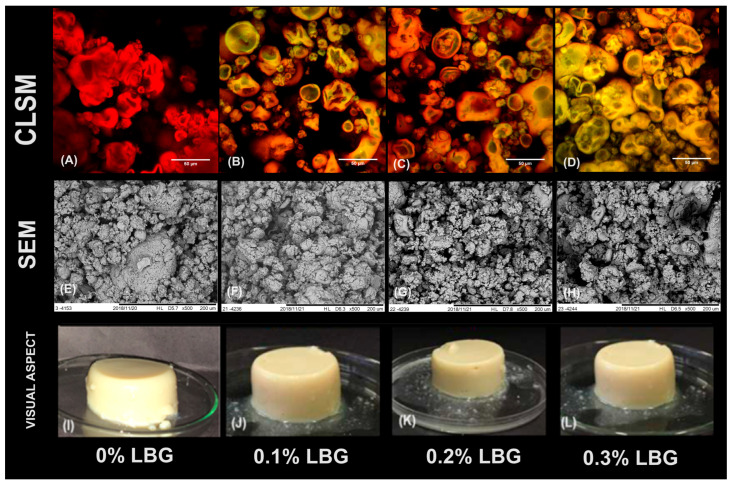
Confocal laser scanning micrographs (CLSMs) (in which Red identifies the protein phase; green identifies the polysaccharide phase) (**A**–**D**), scanning electron micrographs (SEMs) (**E**–**H**) and visual aspects (**I**–**L**) of gels produced with different locust bean gum (LBG) concentrations.

To verify the possibility of replacing NaCl by CaCl_2_ as a gelling agent, the same SPI concentration (14%) and ionic strength (µ: 300) applied in our previous investigation using NaCl was tested with the divalent salt (CaCl_2_). As verified in Figure 1I, the formulation tested resulted in the formation of an opaque self-supported gel, with an opaquer visual aspect in comparison to the systems previously produced using NaCl as a gelling agent [7]. According to the literature, the visual aspects of gelled systems are important to give some valuable information about the nature of protein aggregates, these being directly related to the type of matrix formed [30,31]. In the present investigation, for example, the opaque aspect of the gelled systems resulted from the particulate, disorganized, and porous microstructures, verified in the SEM micrograph Figure 1E, which generally result from a large and random aggregation process [30,31]. From Figure 1E, it was also possible to verify the formation of structures similar to “capsules” in a given region (highlighted in red). Such microstructural organization may be justified by three main factors: (I) the gelation method, involving direct salt addition; (II) the type of salt applied and (III) the mechanisms involved in the gelation of soy proteins.

It is known that direct salt addition in cold-set methods may cause an excessively fast gelation process, resulting in random aggregations which generally lead to the formation of weaker matrices, with less ordered microstructures, such as the one shown in Figure 1E [32,33]. In terms of the type of salts, according to the literature divalent salts (i.e., CaCl_2_) tend to cause much faster and effective interactions among protein aggregates in comparison to monovalent salts (i.e., NaCl), especially due to their higher capacity to screen electrical charges and to form cross-links among negatively charged carboxyl groups [7,30].

The formation of “capsule-like” structures in some regions of the gels, in contrast, possibly resulted from the aggregation processes that occurred during preheating of the SPI (rich in β-conglycinin and glycinin) and subsequent CaCl_2_ addition. In their study, Guo et al. [34] observed that when β-conglycinin is heated in the presence of glycinin at pH 7 (i.e., the pH applied in the gels produced in the present investigation), β-conglycinin’s hydrophilic groups tend to occupy the surface of the complex aggregates formed mainly by glycinin. In this context, it is possible that during the preheating, the aggregation process described by Guo et al. [34] led to an initial formation of the “capsule-like” structures verified in Figure 1E. Considering that the protein concentration used was below the critical concentration for the formation of heat-induced gels [7], such aggregates were still dispersed and only formed a gelled continuous matrix after the incorporation of salt. The microstructural organization observed resembles the ones referred to as microgels by some authors [35]. According to Nicolai and Durand [35], during primary protein aggregation, a finite size of aggregates is formed with, basically, two distinct morphologies: thin elongated aggregates and roughly spherical aggregates, which have also been called microgels (fractal, self-similar, branched aggregates or clusters), implying that the particle (in the present study referred as a “capsule”) is formed by a crosslinked network and contains a relatively large fraction of solvent [35].

**Figure 2:**
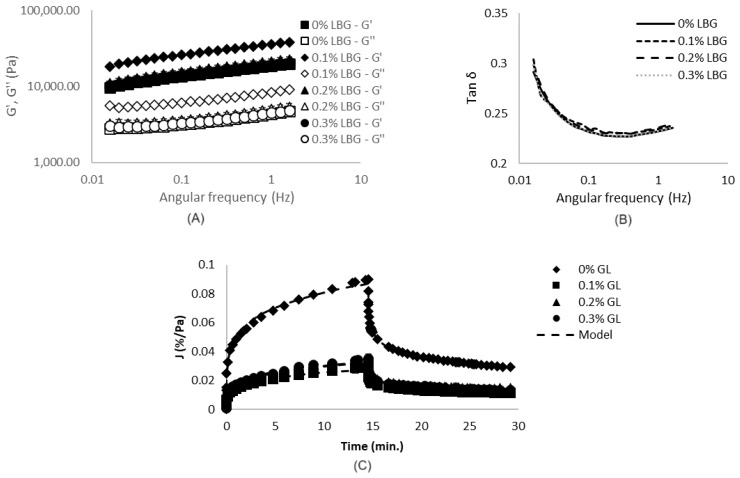
Results of frequency sweep (**A**,**B**) and creep/recovery tests (**C**) of gels produced with different locust bean gum (LBG) concentrations.

The stronger aspect of CaCl_2_-induced gels was confirmed by the rheological data, presented in Figure 2 and Table 1. It was verified that NaCl-induced gels [7] were weaker than CaCl_2_-induced ones (Table 1), presenting smaller values of G′, G″, K′ and K″. In addition, CaCl_2_-induced gels presented higher n′ and n″. Such behavior is related to the fact that, even though both monovalent and divalent salts are able to screen electrostatic interactions between charged protein molecules, resulting in decreases in electrostatic repulsions and protein aggregation, only the divalent cations can act as bridges between the negatively charged carboxylic groups on neighboring protein molecules, directly stabilizing the interactions between them [36,37]. Possibly due to the formation of these salt bridges, CaCl_2_-induced gels presented G′ and G″ with higher frequency dependence in comparison to the reported NaCl-induced gels, indicating a higher contribution of physical interactions in these systems [38]. Additionally, tan δ values were higher and more frequency-dependent for these CaCl_2_-induced gels, indicating lower elastic contributions.

The higher strength and lower elasticity of CaCl_2_-induced gels in comparison to NaCl-induced ones may be explained by the microstructural organization of the systems produced with the divalent salt, which revealed to be much more particulate, as previously verified and discussed from Figure 1E [7].

Such conclusions were confirmed by the results of creep/recovery tests (Figure 2C), which indicated that CaCl_2_-induced gels presented higher hardness and lower elasticities and abilities to recover after stress removal in comparison to NaCl-induced gels [7]. It is possible that, for CaCl_2_-induced gels, the stress application caused disruptions in the salt bridges initially formed between the charged carboxylic groups, reducing the systems’ ability to recover when the stress was removed, in comparison to NaCl-induced systems.

The uniaxial compression tests, on the other hand, showed that CaCl_2_-induced gels presented a higher Young’s modulus and rupture stress in comparison to the reported data for NaCl-induced gels [7]. Rupture strain, however, was less affected, as significant differences (*p* < 0.05) were only verified for systems with 14% SPI at ionic strength of 300 (100 mM CaCl_2_), in which the incorporation of CaCl_2_ resulted in the formation of structures with higher rupture strains.

Even though the obtained gel presented a self-supported aspect, the system exhibits a low water-holding capacity (40.0% ± 2.2). In general, particulate and porous gelled matrices, such as the ones observed in the present study, tend to present low WHC, especially for the lower capacities to retain water by capillary forces [39]. Besides it is known that divalent cations tend to form bridges among between biopolymers chains and, therefore, decrease the amount of hydrophilic groups available to interact with water, which remains free in the pores of the network.

### 2.2. Effects of the Incorporation of Different Locust Bean Gum in CaCl_2_-Induced Gels

The low water-holding capacity is a potential problem in the development of food gels and highlights the challenging properties of the SPI. As previously cited, however, the incorporation of polysaccharides is a potential alternative to improve the gelling capacity of protein ingredients [7,40]. In this context, different LBG concentrations were incorporated to CaCl_2_-induced gels and the systems were characterized, initially in terms of its microstructural organizations (Figure 1F–H). It was verified that the higher the amount of LBG, the higher the heterogeneity of the systems. The increase in polysaccharide concentration up to 0.2% led to the formation of smaller and more separated aggregates. Systems containing 0.3% LBG, however, presented some regions with a more compact aspect, mainly formed by microgel-like structures.

CLSM micrographs (Figure 1B–D) revealed that the formulations with 0.1% and 0.2% LBG presented a certain degree of phase separation, with some reddish and others greenish regions, while systems containing 0.3% LBG presented a lower extension of such phenomenon. According to the literature, thermodynamic incompatibility and phase separation are very commonly observed phenomena in aqueous mixtures of protein and neutral polysaccharides. However, when gelation occurs, the process of phase separation tends to slow down or eventually stop, with the resulting microstructures being “arrested” within the formed polymer network, as verified from the incomplete demixing processes verified, especially at higher LBG concentrations [40].

As expected, such microstructural organizations reflected in the rheological properties of the systems, shown in Figure 2 and Table 1. The incorporation of 0.1% LBG resulted in the strengthening of the matrices, as verified through the increases in G′ and G″ (and, therefore, K′ and K″). The addition of higher LBG concentrations, however, did not significantly change (*p* > 0.05) the values of K′ and K″ in comparison to SPI only gels. For all formulations, on the other hand, the values of tan δ, n′ and n″ were similar.

The results of the creep/recovery tests (Figure 2C), however, indicated a decrease in compliance values for all three LBG concentrations tested. Such results revealed that, even though systems with 0.1% LBG presented a higher strength under small deformations, the presence of such galactomannan increased the resistance of the gels to deform under the application of the constant stress during the creep step, resulting in decreases in J_0_ and J_1_, in comparison to SPI-only gels. In addition, LBG-SPI mixed gels presented higher η_0_. λ_ret_ and the recovery capacities of the systems, on the other hand, were not significantly affected.

The strengthening verified through the incorporation of LBG (especially at 0.1%) is related to the increase in protein–protein interactions (mainly through saline bridges between charged carboxylic groups) and the higher self-association of polysaccharides, which possibly reduced the effects of incomplete demixing, and resulted in the more evident microphase separations previously discussed.

Uniaxial compression data (Table 1), on the other hand, revealed that the LBG incorporation decreased E_g_ in all concentrations and decreased the rupture parameters in concentrations equal or higher than 0.2%. Therefore, even though LBG incorporation increased the strength of the gels under oscilatory/small deformations, it compromises system strength under large deformations, possibly due to the heterogeneous and more porous microstructures previously discussed. According to the literature, even though microphase separations may increase interactions among biopolymers within each phase, and also the elastic response of systems, such effect depends on the connectivity between protein aggregates [7,41]. Therefore, it was possibly the fragile connectivity within the systems with higher LBG concentrations (0.2 and 0.3%) resulted in the lowest rupture parameters verified. Such fragility, however, did not compromise the strength of the systems under smaller deformations, as verified from frequency sweep tests and creep/recovery tests.

Regarding the water holding-capacity, systems with 0.1% LBG presented the higher values, with an average of 46.5% ± 0.4, followed by 0.2% LBG (WHC: 45.0% ± 1.5) and 0.3% LBG (42.1% ± 2.2). Observing the results obtained in this section, it is clear that the formulation with 0.1% LBG was the most promising, with a relatively higher degree of phase separation, higher WHC and higher resistance in terms of rupture properties under large deformations and general higher resistance under small deformations. Therefore, such formulation was selected to the next step of this investigation, which consisted in evaluating the stability of the gels.

### 2.3. Evaluation of Gel Stability

**Figure 3:**
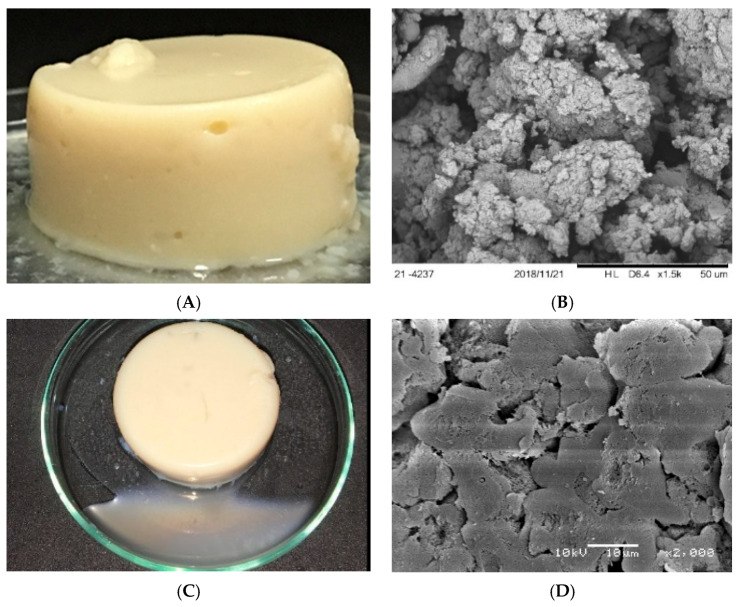
SEM micrographs and visual aspects of non-filled gels at days 1 (**A**,**C**) and 10 (**B**,**D**) of storage.

The quantitative prediction of gelled systems’ stability is considered a critical issue in the formulation of commercial food products [25], for this reason the formulation produced with using SPI (14%), LBG (0.1%) and CaCl_2_ (100 mM) was evaluated during storage. For the first day, all the characterization data previously discussed were considered and, therefore, the gels were self-supported and presented an opaque aspect (Figure 3A). Even though the system presented a relatively low WHC (46.5% ± 0.4), the gel could be adequately analyzed through frequency sweep tests, creep/recovery tests and uniaxial compression tests, as previously shown in Figure 2 and Table 1. After 10 days, however, the excessive syneresis verified in Figure 1C prevented the development of rheological analyzes for compromising the accommodation of the samples in the equipment. For this reason, at the 10th day of storage, the gels were only analyzed regarding the visual aspect (Figure 1C) and microstructural organization (Figure 1D). The micrograph evidenced that the aging process led to a significant compaction in the matrices’ microstructures, which led to the expulsion of the water initially trapped in the gels’ pores (extensive syneresis) and prevented further characterizations in subsequent days. According to Alting et al. [38], open clusters of aggregates (similar to the ones verified at day 01) are thermodynamically unstable and can be partly stabilized by the formation of additional bonds, which causes the compaction verified at the 10th day of storage (Figure 1D).

According to Barlett et al. [25], a gel is a metastable phase with a high free-energy density whose consolidation is driven by a force for phase separation. When the network is formed, however, the dynamics of phase separation are slowed down. With ageing, the network lowers its free energy via structural reorganizations which proceed through the rupture of single-particle bonds, diffusion to denser region of the network, and a reformation of broken bonds, with a net increase in the number of nearest-neighboring particles [25]. Such processes tend to make the network coarsen, as verified in the present investigation.

Even though the tendency of the gelled systems is to suffer microstructural rearrangements to low free energy, it is known that the velocity through which these alterations will occur up to a point of compromising a food products acceptability can be altered by formulation changes. For this reason, solid lipid microparticles were incorporated to the gels as a possible strategy to improve their stability, decreasing the undesirable changes, which led to the excessive water loss from the matrices, and also for opening a possibility for future bioactive incorporations to the formulation.

### 2.4. Incorporation of Solid Lipid Microparticles as a Strategy to Improve the Gels’ Stability/Shelf-Life

**Figure 4:**
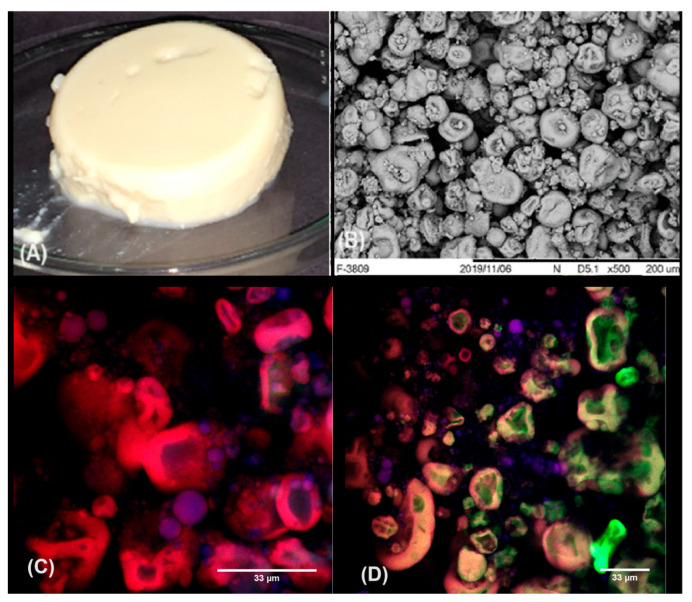
Visual aspect (**A**), scanning electron micrographs (SEMs) (**B**) and confocal laser scanning micrographs (CLSMs) (**C**,**D**) of emulsion-filled gels at the first day of storage.

Solid lipid microparticles were, then, incorporated to the gels and the obtained emulsion-filled gels (EFGs) were initially characterized in terms of their visual aspect, microstructural organization and water-holding capacity on the first day of storage, and the obtained data are shown in Figure 4. The EFGs were self-supported gels (Figure 4A), with larger aggregates/structures which resembled capsules or microgels (Figure 4B), in comparison to the non-filled systems. As verified in Figure 1C,D, the presence of SLMs decreased the extent of incomplete demixing, increasing the microphase separations in the micrographs. Considering that the microstructural organizations of such mixed gels result from the competition between gelation and the phase separation process [40,41], and also that the salt concentration and incorporation methods were the same for non-filled gels and EFG, it is hardly possible that the SLMs affected the gelation rate. However, the presence of SLMs probably increased the rate of phase separation, due to the low chemical affinity between the protein ingredient and the surfactants Tween 80 and Span 80, which increased the thermodynamical incompatibility within the gels and accelerated the phase separation process [28,29].

In some studies found in the literature, the authors affirm that phase separation in protein–polysaccharide mixed gels may increase local biopolymer concentrations in each phase, which favors a more extensive polymer self-association, with potential increases in the gels’ strength [40]. In the present investigation, however, such an effect was not verified, as shown in Table 2. The results demonstrated that the incorporation of SLMs to the gels decreased their strength, as verified from the lower values of E_g_ and rupture parameters of the EFGs in comparison to non-filled gels.

In this case, the rheological data may also be explained by their microstructural organization. Possibly, before the gelation process, the molecules of proteins and polysaccharides were confined in some regions of the samples as a result of the repulsive interactions between the Tween 80/Span 80-stabilized SLMs and biopolymers. With CaCl_2_-incorporation, however, an extensive polymer self-association took place in punctual regions of the systems (in which the biopolymers were concentrated), leading to the formation of the “capsules/microgel”-like structures. Although the microphase separation was more intense within the microgels, the connections among these individual structures were potentially compromised by the presence of the SLMs (located in the spaces among the microgels as shown in Figure 4C,D), which made the matrix more heterogeneous, discontinuous and, consequently, weak.

On the other hand, EFGs presented higher WHC in comparison to non-filled gels (Table 2), also due to the microstructural organization composed by the microgels. Considering that microgels are generally formed by a crosslinked network and contain a relatively large fraction of solvent inside, it is possible that the increase in water retention in EFGs was more related to the amount of liquid entrapped within the microgels than in the spaces among them [35]. Otherwise, the variations in the spaces among these structures would have caused higher WHC variations, due to distinct capillarity forces.

**Figure 5:**
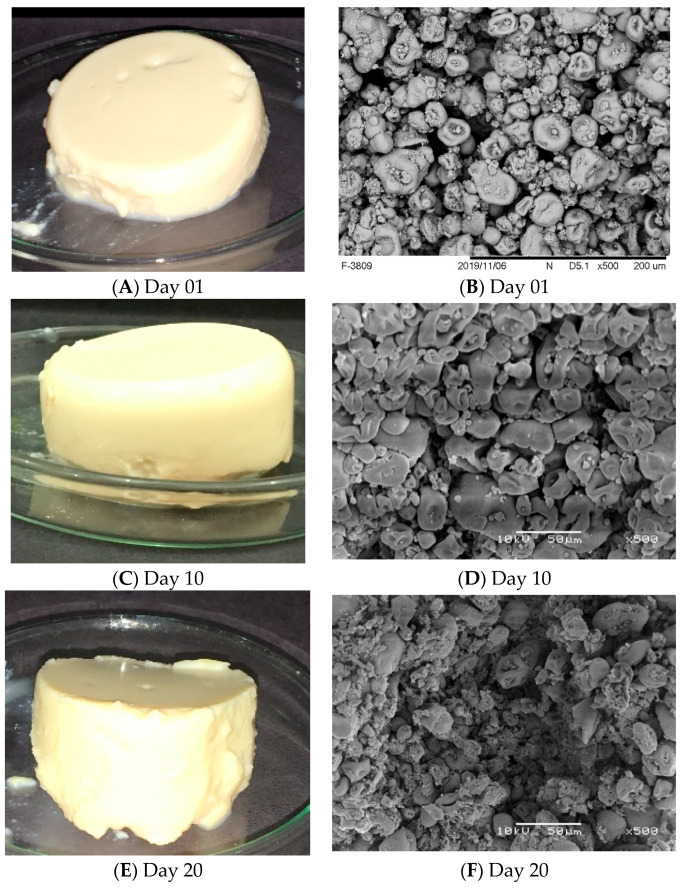
Visual aspects and SEM micrographs of EFGs at different days of storage.

After this initial characterization, the EFGs were stored and evaluated during a 20-day storage period and the results are shown in Figure 5. The visual aspects of the gels on the 10th and 20th day of storage evidenced that the presence of SLMs decreased the loss of water from the gelled systems during storage. Noticeably, the EFGs did not present syneresis during the 20 days analyzed. After this period, however, the EFGs started to lose water and presented microbial growth, which limited the analyzes in subsequent days of storage.

The cited reduction in water loss in the EFGs is related to the microstructural organizations, also shown in Figure 5. Even though the ageing process of the EFGs also led to a microstructural compaction, it was much more subtle than that observed in non-filled gels, indicating that the free energy of the systems containing the SLMs (mainly formed by microgels) was lower than that of particulate non-filled gels. Considering the forms and sizes of microgels were very similar during storage, only with different proximities, it is possible that the ageing process only involved the formation of new bonds among the microgels, and not the breakup of existing bonds within the structural units.

The higher stability of EFGs was also confirmed through the WHC values, which remained constant during the 20 days analyzed (Table 2). Such data reinforced the fact that the larger amount of liquid in the EFGs was entrapped inside the microgels and not among them, not being largely affected by the compaction of the systems, which brought the microgels closer to each other.

**Figure 6:**
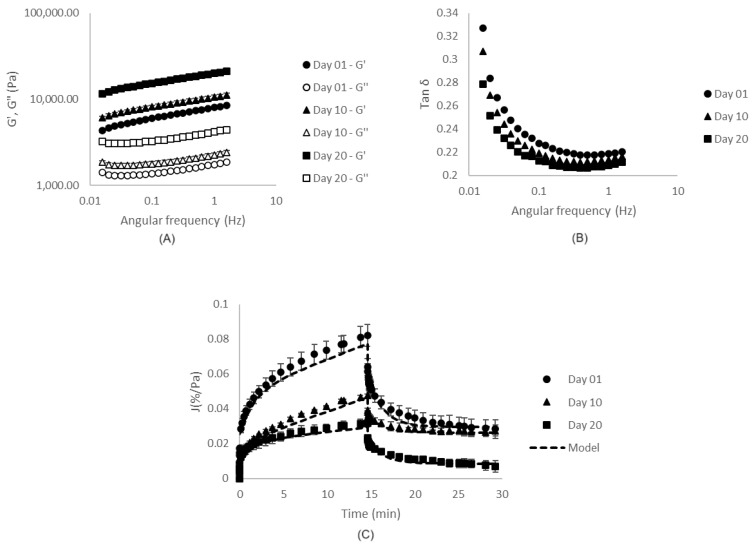
Results of frequency sweep tests (**A**,**B**) and creep/recovery tests (**C**) of EFGs on different days of storage.

Such higher WHC values resulted in a better accommodation of the EFGs in the rheometer allowing the characterization of such systems in the different days of storage. The obtained results are shown in Figure 6 and Table 2. Frequency sweep tests revealed a continuous increase in G′, G″, and, consequently, K′ and K″, and a decrease in n′ during the 20 days of storage. Such strengthening of the EFGs revealed that, for such systems, the ageing process led to the formation of more stable bonds between protein groups reducing the free energy and increasing the microstructural compaction. In addition, it was verified a continuous decrease in the values of tan δ (Figure 6B), indicating an increase in the elastic responses of the EFGs, possibly due to the more effective interactions of the biopolymers after the compaction of the microstructures.

Such alterations also led to decreases in compliance (Figure 6C), J_0_ and J_1_, and increases in ɳ_0_ (Table 2). Interestingly, on the 10th day of storage, the systems had the lowest values of λ_ret_ and recovery capacity, indicating that, although new connections and interactions were established, they were still fragile. On the 20th day, however, such interactions had become more stable, resulting in the further increases in λ_ret_ and recovery capacity.

The strengthening (in this case, increased rigidity) of the EFGs during storage was confirmed by uniaxial compression data, which showed increases in all parameters after the 10th day of storage. Such results confirmed that the incorporation of SLMs was beneficial to the gels’ stability as it decreased the microstructural alterations; however, the rheological properties of the gels were significantly altered. The influences of these changes in the sensory perception of a future food originating from these EFGs is difficult to predict. However, these data allow us to affirm that the incorporation of SLMs was an important step towards the reduction of water loss from the non-filled gels.

## 3. Conclusions

CaCl_2_-induced gels presented particulate, disorganized, and porous matrices, which led to low WHC values. The obtained systems were stronger (higher G′, G″, Eg and rupture properties, and lower compliance values) in comparison to NaCl-induced gels produced in a previous investigation. The low WHC was improved with LBG incorporation, especially in concentrations of 0.1%. In such a concentration, the polysaccharide incorporation increased G′ and G″, indicating a strengthening of the matrices, but decreased E_g_, due to the heterogeneous and more porous microstructures, with incomplete demixing. Gels with SPI (14%), LBG (0.1%) and CaCl_2_ (100 mM) had their properties evaluated during storage, and presented very poor stability, as after 10 days the systems could not be adequately analyzed using a rheometer due to the excessive syneresis resulting from a significant microstructural compaction. The incorporation of solid lipid microparticles to such gels, however, was revealed to be an interesting strategy to improve the stability of the obtained plant-based gel rich in calcium, especially by reducing the microstructural compaction and, therefore, syneresis, for more than 20 days. Even though the rheological properties of the EFGs were significantly altered due to the ageing process (which may affect the sensory perception of a future food originated from such EFGs), the incorporation of solid lipid microparticles increased the systems’ potential to be applied as a product prototype, for even for future incorporations of hydrophobic bioactives.

## 4. Materials and Methods

### 4.1. Chemicals and Reagents

For gel production, the SPI (Protimarti M-90, 84.3% protein) was purchased from Marsul (Montenegro, RS, Brazil), calcium chloride dihydrate from Kyma (Americana, SP, Brazil), and locust bean gum (Viscogum LBG^®^) has been donated by Cargill (Mairinque, SP, Brazil). For SLM production, palm stearin (PS), with melting point of 50.1 °C was donated by Agropalma (Belém, PA, Brazil), while Tween 80 and Span 80 were purchased from Sigma-Aldrich (St. Louis, MO, USA). In all experiments, the water applied was Ultrapure obtained from a Millipore system Direct Q3^®^ (Billerica, MA, USA).

### 4.2. Production and Characterization of Cold-Set CaCl_2_-Rich Gels

The production of cold-set SPI gels was performed using a protocol defined in a previous study [7]. For this purpose, a dispersion of 14% (*w*/*v*) SPI, with pH 7, was produced using deionized water at room temperature. Afterwards, the samples were preheated in a water bath at 80 °C for 30 min and cooled to room temperature. Subsequently, the systems were added with 100 mM CaCl_2_ (i.e., 11.098 mg/mL). Such a salt concentration was calculated to achieve the same ionic strength previously applied to producing NaCl-induced gels [7]. The samples were, then, stored at 10 °C for 12 h before the characterizations.

For the production of SPI-LBG mixed gels, the same protocol was applied, however, but the locust bean gum (LBG) (0.1–0.3%, *w*/*v*) was added to the SPI powder before hydration. The different formulations were characterized in terms of visual aspects, water-holding capacities, microstructural properties (confocal laser scanning microscopy and scanning electron microscopy) and rheological parameters (uniaxial compression tests, small-amplitude oscillatory shear tests and creep/recovery tests).

### 4.3. Production of Emulsion-Filled Gels

#### 4.3.1. Production of Solid Lipid Microparticles (SLMs)

The production of SLMs was performed according to a protocol described by Brito-Oliveira et al. [28]. For this purpose, the lipid phase (composed by palm stearin—4.5%, *w*/*w*, and Span 80—2.7%, *w*/*w*), was heated to 80 °C for 30 min to eliminate the thermal memory. Afterwards, the aqueous phase (composed by Tween 80—1.8%, *w*/*v*—and deionized water) was dispersed in the lipid phase, using a rotor–stator device (IKA T25, IKA, Staufen, Germany) at 18,000 rpm for 5 min, at 80 °C. The systems were added with sodium benzoate (0.02%, *w*/*w*) to avoid microbiological contamination. The solid lipid microparticles dispersions (SLMDs) were stored at 10 °C overnight before their application to produce EFGs.

#### 4.3.2. Production of Emulsion-Filled Gels

For EFG production, the same protocol described in item 2.2 was applied, using a formulation of 14% (*w*/*v*) of SPI, 0.1% (*w*/*v*) of LBG and 100 mM of CaCl_2_; however, 50% of the deionized water used to hydrate the SPI/LBGs was replaced by solid lipid microparticle (SLM) dispersions. The SLM concentration was selected after a preliminary scanning and observation of the visual aspect of the obtained gels. The systems were also stored at 10 °C for 12 h before characterization. The systems were characterized in terms of visual aspects, water-holding capacities, microstructural properties (confocal laser scanning microscopy and scanning electron microscopy) and rheological parameters (uniaxial compression tests, small-strain oscillatory tests and creep/recovery tests).

### 4.4. Evaluation of Gel Stability

For the evaluation of the gels’ stability, non-filled gels and emulsion-filled gels went through evaluations of visual aspects, WHC, morphology (by scanning electron microscopy) and rheological parameters (by uniaxial compression, small-amplitude oscillatory shear and creep/recovery tests) in different days of storage.

### 4.5. Confocal Laser Scanning Microscopy

Confocal laser scanning microscopy (CLSM) (Confocal Upright Microscope LSM 780 NLO-Zeiss, Zeiss, Germany) was conducted according to protocol described by Brito-Oliveira et al. [29], using simultaneous dual-channel imaging. The protein phase was visualized by exciting rhodamine B (stock solution of 0.2%, *w*/*v*, in deionized water—application of 10 μL of stock solution/mL of gel) at a wavelength of 543 nm and at an emission wavelength range of 551–655 nm. The polysaccharides were visualized by exciting the dye fluorescein isothiocyanate (FITC) (stock solution of 1 mg/mL in dimethyl sulfoxide—application of 0.05 mL of stock solution/mL of gel) at an excitation wavelength of 488 nm and an emission wavelength range of 493–543 nm. The SLMs were visualized by exciting the Nile Red dye (stock solution of 0.1 g/100 mL in methanol—application of 10 µL of solution/g of lipid) at an excitation wavelength of 488 nm and an emission wavelength range of 500–580 nm.

### 4.6. Scanning Electron Microscopy

The scanning electron microscopy (SEM) was performed according to a protocol described by Picone, Takeuchi and Cunha [42]. For sample preparation, the gels (1.0 × 0.5 × 0.5 cm) were fixed in 2.5 g/100 g glutaraldehyde in a cacodylate buffer (16 g/L) at pH 7.2 and stored at 7 °C for 24 h. Subsequently, the systems were rinsed twice in a cacodylate sodium buffer (16 g/L, pH 7.2) and fractured in liquid nitrogen. Then, the samples were subjected to post-fixation using osmium tetroxide 1 g/100 g in a cacodylate buffer (16 g/L, pH 7.2) for 120 min and rinsed twice in deionized water. Afterwards, the gels were dehydrated in a graded ethanol series (30, 50, 70 and 90 mL/100 mL) for 20 min in each, and in 100% ethanol (three changes in 1 h). The dehydration was completed by critical-point drying (CPD03 Balzers Critical Point Dryer, Alzenau, Germany). The samples were, then, fractured, placed in aluminum stubs, coated with gold (200 s/40 mA) in a Balzers SCD 050 Sputter Coater (Alzenau, Germany), and analyzed using a TM 3000 tabletop microscope (Hitachi, Tokyo, Japan).

### 4.7. Water-Holding Capacity

The water holding capacity (WHC) of the gels was analyzed using protocol described by Beuschel, Culbertson, Partridge and Smith [43]. For this purpose, the systems were weighted on Whatman paper No. 1 placed in Falcon tubes and centrifuged at 2500 rpm for 10 min at 6 °C (Hermle centrifuge Labortechnik GmbH, model Z-216 MK, Wehingen, Germany). Subsequently, the samples were removed from the filters, which were weighted for the determination of the water mass released. The values of WHC were calculated using Equation (1).

(1) $$WHC\left(\%\right)=\left(1-\left(\frac{m_{f}-m_{i}}{m_{s}}\right)\right)\times 100$$

where *m_f_* is the weight of the wet filter (g), m_i_ is the initial weight of the dry filter (g), and *m_s_* is the weight of the SPI sample (1–2 g). Each experiment was performed in triplicate.

### 4.8. Uniaxial Compression

For the development of uniaxial compression tests, a protocol adapted from Oliver, Scholten and van Aken [44] was applied, using a texturometer (TA-XT.plus Texture Analyser, Godalming, Surrey, UK). For this purpose, the gels with a cylindrical shape were compressed to 80% of their original height using an aluminum probe lubricated with silicone oil to minimize friction. The formulations were tested using five replicates and a deformation speed of 1 mm/s. Hencky stress (σ*_H_*) and Hencky strain (ε*_H_*) values were calculated from the force–deformation data using Equations (2) and (3), respectively:

(2) $$\sigma_{H}=F\left(t\right)\cdot \frac{H(t)}{H_{0\;}\cdot \;A_{0}}$$

(3) $$\varepsilon_{H}=\ln\frac{H(t)}{H_{0}}$$

where F(t) is the force at time *t*, *A*_0_ is the initial area, *H*_0_ is the initial height, and *H*(*t*) is the height at time *t*. The rupture parameters were associated with the maximum value of the stress–strain curve. The values of the apparent Young’s modulus (E_g_) of the systems were determined by the slope of the first linear interval in the Hencky stress (σ*_H_*) versus Hencky strain (ε*_H_*) curves, up to rupture.

### 4.9. Small Strain Oscillatory Tests

Small amplitude oscillatory shear tests were developed using protocol adapted from Chang, Li, Wang, Bi and Adhikari [45]. The tests were performed in an AR2000 rheometer (TA Instruments, New Castle, DE, USA), at 10 °C, with aluminum parallel plate geometry (60 mm diameter, 1 mm gap). Silicone oil was used at the edge of the samples to avoid water evaporation, and a 2 min resting period was applied before each experiment. The linear viscoelastic region (LVR) of each sample was determined through strain sweep tests, which were developed in the range 0.01–100%, using a constant frequency of 1 Hz. Frequency sweep tests, on the other hand, were carried out over an angular frequency range of 0.016–1.6 Hz, using a strain amplitude of 2% (determined for being within the LVR). The angular frequency dependence of both viscoelastic moduli (*G*′ and *G*″) was described by a power law model (Equations (4) and (5)), using the nonlinear regression feature in Excel (Microsoft, Seattle, WA, USA) [45]:

(4) $$G^{\prime }=K^{\prime }\cdot \omega^{n\prime }$$

(5) $$G^{\prime \prime }=K^{\prime \prime }\cdot \;\omega^{n\prime \prime }$$

where *K*′ and *K*″ are power law parameters, *n*′ and *n*″ are frequency exponents, and ω is the angular frequency.

### 4.10. Creep/Recovery Tests

For creep/recovery tests, a protocol described by Brito-Oliveira et al. [7] was applied and the experiments were developed in an AR2000 rheometer (TA Instruments, New Castle, PN, EUA), using an aluminum parallel plate geometry (60 mm diameter, 2 mm gap), at 10 °C. A resting time of 10 min was applied for the elimination of loading effects. For the creep step, a constant stress of 5 Pa was applied for 15 min and then removed for the evaluation of the recovery behavior of the samples for another 15 min. Silicone oil was also used on the edges of the samples to avoid water evaporation. Creep and recovery data were analyzed using the four-parameter Burger’s model represented by Equation (6) [45].

(6) $$J\left(t\right)=\left\{\begin{array}{c} J_{0}+J_{1}\left(1-\exp\left(\frac{-t}{\lambda_{ret}}\right)\right)+\frac{t}{{ɳ}_{0}},\;t\leq t_{1}\\ J_{1}\left(\exp\left(\frac{t_{1}-t}{\lambda_{ret}}\right)-\exp\left(\frac{-t}{\lambda_{ret}}\right)\right)+\frac{t_{1}}{{ɳ}_{0}},\;t>t_{1} \end{array}\right.$$

where *J*_0_ is the instantaneous compliance in %/Pa, ɳ_0_ is the viscosity of the Maxwell dashpot in Pa·s/%, *J*_1_ is the compliance associated with the Kelvin–Voigt element in %/Pa, *λ_ret_* is the retardation time associated with the Kelvin–Voigt element in s, and *t*_1_ is the time when the stress was removed.

The recovery rates were calculated using Equation (7), where *ε_max_* is the maximum strain at the end of the creep test and *ε_f_* is the final strain [45].

(7) $$Recovery\;\left(\%\right)=\left(\frac{\varepsilon_{\max}-\varepsilon_{f}}{\varepsilon_{max}}\right)*100$$

### 4.11. Statistical Analyses

All measurements were performed at least in triplicate, and mean values and corresponding errors were calculated. For the statistical treatment of data, an analysis of variance (ANOVA) was conducted, followed by Tukey’s tests with a 5% significance level using SAS software version 9.2 (SAS Institute Inc, Cary, NC, USA).

## Figures and Tables

**Table 1:** Parameters of Power Law model (frequency sweep data), Burger’s model (creep/recovery data) and uniaxial compression data of SPI gels produced with different locust bean gum (LBG) concentrations.

Analysis	Parameter	LBG Concentration			
		0%	0.1%	0.2%	0.3%
Frequency sweep	K′ (Pa)	18,881 ^b^ ± 1109	36,225 ^a^ ± 1750	21,322 ^b^ ± 1865	19,550 ^b^ ± 1072
	n′	0.150 ^a^ ± 0.003	0.151 ^a^ ± 0.002	0.149 ^a^ ± 0.002	0.146 ^a^ ± 0.002
	R^2^	0.99	0.99	0.99	0.99
	K″ (Pa)	4233 ^b^ ± 248	8208 ^a^ ± 401	4852 ^b^ ± 433	4376 ^b^ ± 263
	n″	0.115 ^a^ ± 0.004	0.117 ^a^ ± 0.001	0.115 ^a^ ± 0.003	0.112 ^a^ ± 0.002
	R^2^	0.96	0.96	0.95	0.95
Creep/recovery	J_0_ (Pa^−1^)	0.031 ^a^ ± 0.001	0.009 ^b^ ± 0.002	0.010 ^b^ ± 0.001	0.011 ^b^ ± 0.002
	J_1_ (Pa^−1^)	0.021 ^a^ ± 0.001	0.006 ^b^ ± 0.002	0.007 ^b^ ± 0.001	0.008 ^b^ ± 0.001
	λ_ret_ (s)	39.58 ^a^ ± 1.32	58.61 ^a^ ± 31.97	55.52 ^a^ ± 5.93	66.05 ^a^ ± 9.72
	ɳ_0_ (Pa.s)	27,865 ^b^ ± 1232	76,547 ^a^ ± 17,173	5995 ^a^ ± 2398	63,553 ^a^ ± 7003
	R^2^	0.88	0.90	0.91	0.91
	Ɛ (%)	68.0 ^a^ ± 1.9	61.7 ^a^ ± 8.6	56.3 ^a^ ± 1.9	62.1 ^a^ ± 2.8
Uniaxial compression	E_g_ (Pa)	4469 ^a^ ± 97	4064 ^b^ ± 82	2877 ^c^ ± 248	2868 ^c^ ± 102
	σ_H_ (Pa)	1301 ^a^ ± 333.9	1519 ^a^ ± 199	845 ^b^ ± 82	859 ^b^ ± 93
	Ɛ_H_	0.288 ^a^ ± 0.046	0.346 ^a^ ± 0.056	0.212 ^b^ ± 0.031	0.225 ^b^ ± 0.014

Averages followed by different lowercase letters are statistically different (*p* < 0.05) for gels with different concentrations of LBG.

**Table 2:** Parameters of Power Law model (frequency sweep data), Burger’s model (creep/recovery data) and uniaxial compression data of emulsion-filled gels (EFGs) at different days of storage.

Formulation	Parameter	Day 01	Day 10	Day 20
Water-holding capacity	WHC (%)	65.0 ^a^ ± 4.6	61.7 ^a^ ± 3.1	63.3 ^a^ ± 0.5
Frequency sweep tests	K′ (Pa)	8103 ^c^ ± 318	10,678 ^b^ ± 569	2011 ^a^ ± 562
	n′	0.1367 ^a^ ± 0.0023	0.1238 ^b^ ± 0.0018	0.1229 ^b^ ± 0.0038
	R^2^	0.99	0.99	0.99
	K″ (Pa)	1698 ^c^ ± 50	2192 ^b^ ± 131	4060 ^a^ ± 92
	n″	0.0754 ^a^ ± 0.0042	0.0710 ^a^ ± 0.0074	0.0805 ^a^ ± 0.0035
	R^2^	0.86	0.82	0.91
Creep/recovery tests	J_0_ (%/Pa)	0.0255 ^a^ ± 0.0022	0.0123 ^b^ ± 0.0003	0.0109 ^b^ ± 0.0017
	J_1_ (%/Pa)	0.0231 ^a^ ± 0.0013	0.0084 ^b^ ± 0.0009	0.0101 ^b^ ± 0.0021
	λ_ret_ (s)	99.06 ^ab^ ± 5.21	89.26 ^b^ ± 9.10	107.73 ^a^ ± 4.67
	ɳ_0_ (Pa.s/%)	30,914 ^b^ ± 4674	34,352 ^b^ ± 2886	110,475 ^a^ ± 29,707
	R^2^	0.91	0.93	0.89
	ε (%)	65.7 ^a^ ± 4.8	42.4 ^b^ ± 5.8	78.0 ^a^ ± 11.6
Uniaxial compression tests	E_g_ (Pa)	3161 ^b^ ± 419	7533 ^a^ ± 436	7857 ^a^ ± 537
	σ_H_ (Pa)	1092 ^b^ ± 72	2115 ^a^ ± 209	2348 ^a^ ± 240
	ε_H_	0.2398 ^b^ ± 0.0097	0.2751 ^ab^ ± 0.0220	0.2956 ^a^ ± 0.0191

Averages followed by different lowercase letters in the same line are statistically different (*p* < 0.05) for EFGs at different days of storage.

## Data Availability

The raw data supporting the conclusions of this article will be made available by the authors on request.
